# Disease-Specific Knowledge, Physical Activity, and Physical Functioning Examination among Patients with Chronic Non-Specific Low Back Pain

**DOI:** 10.3390/ijerph191912024

**Published:** 2022-09-23

**Authors:** Márta Hock, Melinda Járomi, Viktória Prémusz, Zsolt János Szekeres, Pongrác Ács, Brigitta Szilágyi, Zhe Wang, Alexandra Makai

**Affiliations:** 1 Faculty of Health Sciences, University of Pécs, 3 Vörösmarty Str., H-7621 Pecs, Hungary; 2 Division of Preventive Cardiology and Rehabilitation, Medical School, University of Pécs, 2 Rákóczi Str., H-7623 Pecs, Hungary

**Keywords:** physical activity, back school, chronic non-specific low back pain

## Abstract

Physical activity, physical functioning, and pain are some of the most critical factors of low back pain (LBP) treatment and prevention, but it was unknown that the back school program (BSP) influences the physical activity level of the patients with LBP. Data from 306 healthy patients and patients with chronic non-specific low back pain (cnsLBP) were used. We used the Global Physical Activity Questionnaire (GPAQ), the Low Back Pain Knowledge Questionnaire (LKQ), the visual analog scale, and the Roland–Morris Disability Questionnaire (RMDQ). The significance level was set at *p* < 0.05. The amount of sedentary time in cnsLBP patients enrolled in the BSP was significantly lower compared to the other two groups (*p* < 0.001). Significantly higher moderate-intensity activities, leisure time activities, and active transportation were observed in the cnsLBP patients enrolled in the BSP than in the other two groups (*p* < 0.001). RMDQ scores and the pain intensity of the cnsLBP patients enrolled in the BSP were significantly lower than in patients with LBP receiving only exercise therapy (*p* < 0.001). The physical activity level and low-back-pain-specific knowledge was significantly higher, while back-related disability and pain intensity were significantly lower among patients with low back pain syndrome who participated in a back school program.

## 1. Introduction

In Europe, the global prevalence of low back pain (LBP) was estimated to be 13% for males and 10% for females in 2010 [1]. Chronic non-specific low back pain (cnsLBP) syndrome is a multifactorial bio-psycho-social disease. Based on Bálint’s definition, CnsLBP is a set of symptoms related to low back pain, which is characterized by a narrowing of the range of motion (ROM), sensitivity to pressure, antalgic posture, paravertebral spasm, and pain from the 12th thoracic vertebra to the tuber ischiadicum [2]. Non-specific low back pain is usually categorized into three subtypes: acute, sub-acute, and chronic low back pain. This subdivision is based on the duration of the back pain. Acute low back pain is an episode of low back pain for less than 6 weeks, sub-acute low back pain between 6 and 12 weeks, and chronic low back pain for 12 weeks or more [2].

Physical activity also plays a role in pain prevention, rehabilitation, and the prevention of the disease’s relapses [3,4,5]. Inadequate physical activity is one of the causes of cnsLBP. Excessive (sports injuries or exercise addiction) or reduced physical activity (deconditioning) may result in cnsLBP syndrome as well. After the onset of cnsLBP disease, physical activity may be further reduced due to pain, fear of physical activity, or uncertainty on how to apply the different types of physical activity best, because cnsLBP patients have insufficient knowledge about physical activity. Appropriate physical activity and different movement therapies can play a significant role in the treatment of cnsLBP and the prevention of relapses. Proper physical activity in everyday life and sports activity after rehabilitation is a significant issue for patients. CnsLBP patients do not know the required quantity and quality of physical activity during cnsLBP prevention and rehabilitation. Patients’ disease-specific knowledge in terms of physical activity, prevention, and the treatment of the disease is incomplete or inadequate. According to Werber et al., the majority of the German population did not know about the recommended physical activity level and the beneficial effects of exercise on cnsLBP. Only 35.9% of patients held the opinion that physical activity and movement therapy were efficient in cnsLBP, and according to 50% of patients, passive therapies were effective [6,7], although studies published between 1996 and 2000 clearly showed that active therapies are more effective in cnsLBP for the long term. European and American guidelines [7,8] also prefer physical activity during this period. Patients do not have adequate knowledge about the recommended treatment and physical activity principles. According to the results of the LBP knowledge questionnaire [9] for the assessment of cnsLBP-related knowledge about treatment, the performance on the knowledge test is 50% for Hungarian patients [10], 36% for Brazilian patients [9], and 38% for Saudi Arabians patients [11]. This study aimed to assess the physical activity, low-back-pain-specific knowledge, back-related disability, and pain intensity among patients with low back pain syndrome who participated in a back school program or exercise therapy and healthy participants.

## 2. Materials and Methods

### 2.1. Participants

**Figure 1:**
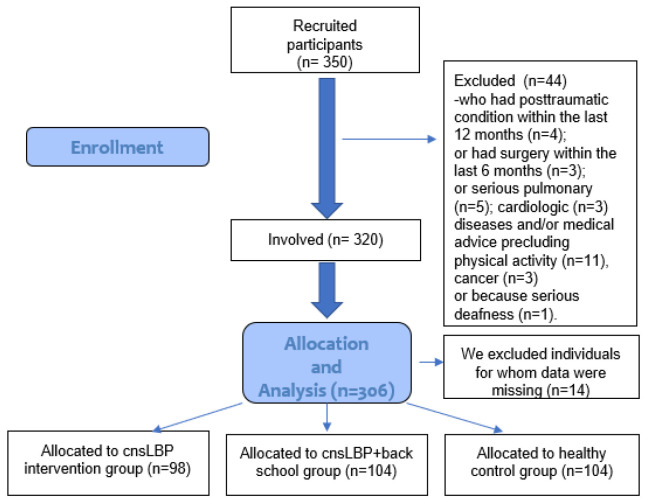
Flow chart of the sampling of the present research.

Overall, 350 participants (healthy and patients with cnsLBP) were recruited, and the cross-sectional survey was finally conducted with 306 participants in Baranya County (Hungary) between January and March 2019. Three groups were studied: (A) healthy participants, (B) cnsLBP patients participating in the back school program, and (C) cnsLBP patients participating in exercise therapy (Figure 1). The inclusion criteria for each group were (A) healthy participants: no history of LBP; no post-traumatic conditions within the last 12 months; no surgery within the last 6 months; for (B) cnsLBP patients in back school program: participation in a 12-week back school program and cnsLBP persisting for at least 3 months; and for (C) patients with cnsLBP syndrome in exercise therapy: cnsLBP patients who participated in 12 weeks of exercise therapy and cnsLBP persisting for at least 3 months as well. The inclusion criterion among all cohorts was age between 18 and 65 years. Exclusion criteria were body mass index (BMI) greater than 35, chronic pain syndrome, depression, non-Hungarian mother tongue, LBP with a non-spine origin (chronic pain syndromes, fibromyalgia, and psychosomatic pain symptoms), and other severe musculoskeletal, neurological, internal, and psychiatric diseases. Two physicians and two physiotherapists were involved. The specialists (a rheumatologist and a neurologist) carried out patient assessment and enrollment into the groups at the Institute of Physiotherapy and Sport Sciences of the University of Pécs. Convenience sampling was used. Elected patients were placed on a waiting list, and when the examined patient numbers reached 6–10, the BS program or exercise intervention began. The healthy participants were enrolled with snowball sampling. Surveys (after acquiring signed informed consent) were conducted in the first and last (12th) weeks.

### 2.2. Exercise Intervention

It was a supervised group exercise intervention. During the exercise intervention, static and dynamic lumbar stabilization and posture correction exercises were used in standing and sitting positions [12]. The exercise intervention was carried out twice a week for 40 min by physiotherapists. During the study, 5 patients dropped out of the exercise intervention group due to deterioration (*n* = 2) or for family (*n* = 2) and work reasons (*n* = 1) (Table 1).

### 2.3. Back School Program

The back school program (BSP) consists of a therapeutic program given to a group of patients with cnsLBP that includes both education and exercise. In the back school education program, cnsLBP patients receive the following information about physical activity: (1) stay active, do their daily activities but more slowly; (2) relative rest, recommended alternation of rest and activities; (3) lying down rather than sitting down at rest; (4) choose physically active leisure and travel activities considering the rules of spine protection; (5) perform the learned exercises 3–7 times a week for 30 min [12,13]. The back school program was carried out twice a week for 60 min by physiotherapists. The physiotherapists involved patients in activities simulating the real situation of their daily living environment (practical training) as well. Two patients dropped out of the BSP group (one person due to deterioration and one person due to family reasons).

In our present study, we analyzed the follow-up scores of the examined patient groups.

### 2.4. Outcome Measures

The Global Physical Activity Questionnaire (GPAQ) was developed by the World Health Organization (WHO) for physical activity surveillance. It collects information with 16 questions (P1–P16) on physical activity participation in three settings (or domains) as well as sedentary behavior. The domains are activity at work, traveling to and from places, and recreational activities. For the three examined domains (activity at work, active travelling, and recreational activities), time (weekly minutes) was calculated according to vigorous-intensity and moderate-intensity activities. The number of days spent active was multiplied by the minutes of the activities [14,15].

Disease-specific knowledge was examined with the Low Back Pain Knowledge Questionnaire (LKQ) [9,10]. The questionnaire consists of 16 simple and multiple choice questions concerning spinal anatomy, biomechanics, the pathophysiological mechanisms of spinal diseases, the prevention and treatment of spinal diseases, and rehabilitation. The questionnaire was evaluated according to international guidelines. We determined the cnsLBP knowledge as points achieved on the questionnaire. The maximum score was 24.

The Roland–Morris Disability Questionnaire (RMDQ) consists of 24 statements relating to the person’s perceptions of their back pain and associated disability [16]. This includes items on physical ability/activity (15), sleep/rest (3), psychosocial factors (2), household management (2), eating (1), and pain frequency (1). It is designed to take approximately 5 min to complete without any assistance from the administrator. The score can range from 0 (no disability) to 24 (maximal disability).

Pain intensity was recorded using the visual analog scale (VAS) with values from 0 (no pain) to 10 (worst possible pain) during regular physical activity in the week before the examination. Participants select the point on the line that best represents their perception of pain intensity level. A higher score indicates greater pain intensity [17].

Age (years) and body mass index (kg/m^2^) of the study groups (healthy controls; cnsLBP; cnsLBP + back school) were assessed in addition to physical activity. Furthermore, we assessed the subjective physical activity status (active versus inactive). Adults who do not do the equivalent of 150 min of moderate-intensity physical activity a week were defined as inactive or insufficiently active [15].

The groups were assessed before (after obtaining written informed consent) and after twelve sessions by physiotherapists during the face-to-face interview.

### 2.5. Statistical Analysis

Data analysis was performed using statistical software IBM SPSS 25.0 (IBM Corp., Armonk, NY, USA). Descriptive statistics were reported for continuous variables as median (interquartile range; IQR). Spearman’s rank correlation and multivariate linear regression were used to determine the association of LBP knowledge and physical activity patterns, age, RMDS, and VAS scores adjusted for the examined groups. Statistical tests were corrected using Bonferroni correction to account for multiple comparisons. The difference between the healthy control group, the LBP patients, and the LBP + back school groups was measured by Kruskal–Wallis and chi-square tests according to the results of the normality test (Kolmogorov–Smirnov tests). The prior target sample size of the study was calculated using G*Power 3.1.9.6 software (Heinrich-Heine-University, Düsseldorf, Germany) for Windows, of which the result was 307 participants [18]. The significance level was set at *p* < 0.05.

## 3. Results

The final sample contained 306 participants: 48.69% of them were male, and 51.31% were female persons.

The mean age of healthy subjects was 42.59 (11.19 SD) years, the mean age of cnsLBP patients enrolled in the back school program was 45.06 (8.02 SD) years, and the time since diagnosis was 4.24 (0.92 SD) years. The mean age of cnsLBP patients receiving exercise therapy was 47.68 (9.20 SD) years, and the time since diagnosis was 4.23 (0.94 SD) years. There was a significant difference regarding age (*p* = 0.017) (Table 2).

### 3.1. Results of the Global Physical Activity Questionnaire

The least amount of sedentary time was found in cnsLBP patients enrolled in the back school program, at 603.75 min/week (*p* < 0.001). No significant difference was found between the groups in the vigorous-intensity activities (*p* < 0.053). Significantly high moderate-intensity activities and moderate leisure time activities were detected in the group of cnsLBP patients enrolled in the back school program (251.1 min/week and 246.44 min/week; *p* < 0.001). A significantly greater amount of physical activity was found during moderate workplace activities in the healthy group at 57.93 min/week (*p* < 0.001). The amount of the cnsLBP patients’ active transportation enrolled in the back school program was significantly higher (39.09 min/week; *p* < 0.001) than in the other two groups (Table 3).

### 3.2. Results of Low Back Pain-Specific Knowledge

Untrained and healthy participants achieved 9.00 points in mean (36.2%) on the test, measuring the knowledge of low back pain. The back school program participants’ performance was 21.00 points (86.9%; *p* < 0.001; Table 4).

### 3.3. Results of Low-Back-Specific Functional Outcome

The cnsLBP patients’ RMDQ score of those enrolled in the back school program was significantly lower (0.04 points) than in patients receiving only exercise therapy (2.8 points; *p* < 0.001; Table 4).

### 3.4. Results of the VAS Scale and Physical Activity Recommendations

The pain intensity of the cnsLBP patients enrolled in the BSP was significantly lower (0.00 points) than in patients participating in exercise therapy (2.00 points; *p* < 0.001; Table 4). It was found that 24.0% of the healthy group, 0.9% of the patients in the exercise program, and 100% of the patients in the BSP met the WHO-recommended physical activity levels (Table 5).

Furthermore, in this study, we examined the effect of low back pain knowledge on physical activity level (dependent variable: total physical activity min/week) by using a multivariate linear regression model. We adjusted the present study results by the Roland–Morris score, age, and VAS values. The results show that low back pain knowledge has a significant role in physical activity level (R^2^ = 0.687; F = 331.826; *p* < 0.001, B = 16.490, *p* < 0.001) and the low-back-specific functional status measured by the Roland–Morris index showed a negative correlation with the physical activity level of the examined patients (B = −19.141, *p* < 0.001).

## 4. Discussion

During the back school program of the present study, patients received theoretical training in addition to education about the pathophysiological mechanism of LBP, preventive activities, the prevention of relapses, the amount and type of recommended physical activity, spine-friendly work and leisure activities, safe exercise rules, and spine-friendly sports as well. During the back school program, patients tried it in practice. Our low back pain knowledge survey showed similar results compared to international research (Table 6).

An earlier systematic review and meta-analysis indicated that exercise, in combination with education, is likely to reduce the risk of LBP [19]. Based on the findings of the present study, the healthy participants had too little and inappropriate knowledge about LBP.

Based on the earlier study, participants with a history of frequent or chronic LBP are more likely to become less active in their leisure time than healthy people [20]. We found significantly higher moderate leisure-time activity in the group of cnsLBP patients enrolled in the back school program than in healthy participants. A moderate to high level of physical activity during leisure time can protect from frequent or chronic LBP, according to a meta-analysis [21].

It is known that patients participating in the back school program regularly perform the learned physiotherapy exercise program, and the tried-and-tested sports therapy exercises learned during the program may evolve into recreational sports later and may be sustained in the long term [22]. This is supported by the results of the current research. Among the cnsLBP patients, the physical activity of the back school group was significantly higher than those who participated only in exercise therapy. As a result of the present study, the cnsLBP group participating in the back school program has achieved the amount of physical activity specified by the professional guidelines. In contrast, the average value of the physical activity of the healthy group does not reach the 150 min/week moderate activity that is recommended. Patients achieving WHO recommendations in leisure-time activity showed a significantly higher health-related quality of life [15].

According to an earlier study, participation in occupational activities, including frequent lifting and a physically demanding workload, was a medium to strong risk factor for LBP [23]. In the present study results, significantly higher moderate workplace physical activity was found in the healthy group than in the educated cnsLBP participants. Probably, participants with LBP reduced the physical demands of their jobs or previously changed their job during the time between diagnosis and back school participation. Preliminary research showed that 6% changed jobs due to LBP [24]. On the other hand, the knowledge acquired during the back school program can help cnsLBP participants in work-related adaptations. The aim of the back school program is to make cnsLBP patients stay active.

The active transportation domain of the GPAQ examines the frequency and duration of walking and cycling. An aim of our study was to measure the association between these activities and LBP. In our research, the time of active transportation of the cnsLBP patients enrolled in the back school program was significantly longer, and in a recent study, walking was inversely associated with the prevalence of chronic back conditions (including LBP) when the analysis was adjusted for age and sex only.

Nowadays, it is well known that physical activity and exercise activate endogenous pain inhibitory mechanisms and lead to a reduction in sensitivity to noxious stimuli (termed ‘exercise-induced hypalgesia’) regardless of the type of physical activity [25]. Based on earlier studies, our back school program included resistance, coordination, and stabilization exercises as well as the educational program because these can have a beneficial effect on pain over other interventions in the treatment of chronic low back pain [26]. Probably, the current study results were due to these two main components, as the cnsLBP patients participating in the back school program had (besides adequate physical activity) lower pain intensity than patients participating only in the exercise intervention.

The present results studied the influence of disease-specific knowledge on physical activity with linear regression analysis, and they prove that the back school program has an influence on the physical activity of cnsLBP patients. In addition, we found that worse low-back-specific functional status measured by the Roland–Morris Disability Questionnaire negatively influenced the physical activity level of the examined patients. Among participants with LBP, the back school program appears to be effective in improving disability as well, which can promote a rise in physical activity.

Several limitations should also be considered when interpreting the results of this study. First, the present study used a relatively small convenience sample. Second, the primary outcome measure of the different parameters could only be measured subjectively (questionnaires) in the groups of patients, which has some limitations. Third, this study did not include complete information from medical records or health examinations about participants. Fourth, there was a significant difference in the average age of the groups, but it is known that the first attack of low back pain typically occurs between the ages of 30 and 50. Fifth, the measures were self-reported, and the different groups were not followed up after the study [27]. The future directions in this research field based on the present study are educational programs for healthy adults and children and cnsLBP patients.

## 5. Conclusions

In measuring healthy adults, cnsLBP patients and patients participating in a back school program, the physical activity level and low-back-pain-specific knowledge were significantly higher, and back-related disability and pain intensity were significantly lower among patients with low back pain syndrome who participated in a back school program.

## Figures and Tables

**Table 1:** Detailed educational program.

Time	Topics of the Educational Program
First month	Spinal anatomy, spinal kinesiology and biomechanics, physiopathology of back disorders, cause of pain
Second month	Rules of spine protection, spine-friendly lifestyle ergonomics in daily living activities, and proper posture (e.g., posture at work, how to lift and transport objects correctly, etc.), LBP prevention options, LBP therapy options
Third month	Spine-friendly leisure, spine-friendly workplace, spine-friendly sports activities, ergonomic practical training

**Table 2:** Basic data of the study participants.

Median (IQR)	Healthy Control (*n* = 104)	cnsLBP + Back School (*n* = 104)	cnsLBP (*n* = 98)	*p* (Kruskal–Wallis Test)
Age (year)	43.00 (37.25–47.50)	43.00 (39.00–49.00)	45.00 (41.00–58.00)	0.017
BMI (kg/m^2^)	25.48 (22.62–27.42)	25.46 (22.62–27.45)	26.88 (22.72–27.45)	0.227
Time spent since the diagnosis (year)	0.00 (0.00–0.00)	4.00 (3.25–5.00)	4.00 (4.00–5.00)	<0.001

**Table 3:** Physical activity patterns of the examined groups, results of the Global Physical Activity Questionnaire.

	Healthy Control (*n* = 104) Mean (SD)		cnsLBP + Back School (*n* = 104) Mean (SD)		cnsLBP (*n* = 98) Mean (SD)		*p* (Kruskal–Wallis Tests)
sitting time (min/day)	681.01	61.16	603.75	13.67	697.79	104.16	<0.001
work total (min/week)	57.93	72.86	4.66	18.24	22.53	51.29	<0.001
active transportation (min/week)	9.59	26.10	39.09	70.84	17.18	47.15	<0.001
total leisure time (min/week)	23.99	50.59	246.44	47.31	100.46	113.49	<0.001
total physical activity time (min/week)	91.51	86.08	290.19	85.51	140.17	132.48	<0.001

**Table 4:** Results of pain intensity, spinal functional status, and disease-specific knowledge.

		VAS	RMDQ	LKQ
Healthy control (*n* = 104)	Median	0.00	0.00	9.00
	IQR lower	0.00	0.00	8.00
	IQR upper	0.00	0.00	9.00
cnsLBP + back school (*n* = 104)	Median	0.00	0.00	21.00
	IQR lower	0.00	0.00	20.00
	IQR upper	0.00	0.00	22.00
cnsLBP (*n* = 98)	Median	2.00	3.00	9.00
	IQR lower	2.00	2.75	8.00
	IQR upper	3.00	3.00	9.00
	*p* value (Kruskal–Wallis test)	<0.001	<0.001	<0.001

**Table 5:** Proportions of the participants who complied with the physical activity recommendation of WHO compared to the physical activity of the study participants.

Healthy Control (*n* = 104)	cnsLBP + Back School (*n* = 104)	cnsLBP (*n* = 98)	*p* (Chi-Square Test)
*n* = 79	*n* = 104	*n* = 1	
75.96%	100.00%	0.96%	<0.001

**Table 6:** Comparison of spine prevention and lumbar pain knowledge.

Authors (Year)	Examined Population	Methods	Results (Points)
Maciel et al. (2009) [9]	60 participants cnsLBP, mean age: 43 years	LKQ	9.8
Kovács-Babócsay et al. (2019) [10]	58 participants, health care workers, mean age: 22	LKQ	18.92
Awwad et al. (2014) [11]	153 participants cnsLBP, mean age: 40.2 years	LKQ	9
Present research	104 participants cnsLBP + back school, mean age: 45.06 years	LKQ	20.87
	98 participants cnsLBP, mean age: 47.68 years		8.74
	104 healthy participants, mean age: 42.59 years		8.79

## Data Availability

Individual anonymized participant data will not be shared. The pooled study data, protocol, or the statistical analysis plan can be shared upon request at alexandra.makai@etk.pte.hu.
